# Pharmacokinetic and metabolic effects of American ginseng (*Panax quinquefolius*) in healthy volunteers receiving the HIV protease inhibitor indinavir

**DOI:** 10.1186/1472-6882-8-50

**Published:** 2008-08-19

**Authors:** Adriana SA Andrade, Craig Hendrix, Teresa L Parsons, Benjamin Caballero, Chun-Su Yuan, Charles W Flexner, Adrian S Dobs, Todd T Brown

**Affiliations:** 1 Division of Infectious Diseases, The Johns Hopkins University, Baltimore, MD 21287, USA; 2 Division of Clinical Pharmacology, The Johns Hopkins University, Baltimore, MD 21287, USA; 3 Department of Human Nutrition, The Johns Hopkins Bloomberg School of Public Health, Baltimore, MD 21205, USA; 4 Tang Center for Herbal Medicine Research, Department of Anesthesia and Critical Care, Pritzker School of Medicine, University of Chicago, Chicago, IL 60637, USA; 5 Division of Endocrinology and Metabolism, Department of Medicine, Johns Hopkins University, Baltimore, MD 21287, USA

## Abstract

**Background:**

Complementary and alternative medicine (CAM) use is prevalent among HIV-infected patients to reduce the toxicity of antiretroviral therapy. Ginseng has been used for treatment of hyperglycemia and insulin resistance, a common side effect of some HIV-1 protease inhibitors (PI). However, it is unknown whether American ginseng (AG) can reverse insulin resistance induced by the PI indinavir (IDV), and whether these two agents interact pharmacologically. We evaluated potential pharmacokinetic interactions between IDV and AG, and assessed whether AG improves IDV-induced insulin resistance.

**Methods:**

After baseline assessment of insulin sensitivity using the insulin clamp technique, healthy volunteers received IDV 800 mg q8 h for 3 days and then IDV and AG 1g q8h for 14 days. IDV pharmacokinetics and insulin sensitivity were assessed before and after AG co-administration.

**Results:**

There was no difference in the area-under the plasma-concentration-time curve after the co-administration of AG, compared to IDV alone (n = 13). Although insulin-stimulated glucose disposal per unit of insulin (M/I) decreased by an average of 14.8 ± 5.9% after 3 days of IDV (from 0.113 ± 0.012 to 0.096 ± 0.014 mg/kgFFM/min per μU/ml of insulin, p = 0.03, n = 11), M/I remained unchanged after co-administration of IDV and AG.

**Conclusion:**

IDV decreases insulin sensitivity, which is unaltered by AG co-administration. AG does not significantly affect IDV pharmacokinetics.

## Background

Dietary supplements, herbs, and vitamins are used widely among HIV-infected patients [1]. However, the safety or efficacy of many of these therapies has not been formally evaluated.

These agents are often used among HIV-infected patients to prevent or treat the adverse effects of antiretroviral therapy [2]. Among these adverse effects are disorders of glucose metabolism, including insulin resistance[3], glucose intolerance [4], and diabetes mellitus[3], which were described soon after the introduction of highly active antiretroviral therapy (HAART)[5]. Although the etiology of these problems is multifactorial [6], exposure to protease inhibitors (PIs) likely contributes directly. Indinavir (IDV), for example, can worsen insulin sensitivity even after a single dose in healthy volunteers[7].

Ginseng is one of the most commonly used herbs in the United States [8] and has long been used for the treatment of hyperglycemia in Traditional Chinese Medicine [9]. Experimental evidence for its efficacy comes from several studies in both animals and humans [10]. In two small clinical trials, *Panax ginseng *[11] and AG [12] given daily over 8 weeks significantly reduced fasting blood glucose and hemoglobin A1c in patients with type 2 diabetes. While the mechanisms by which ginseng affects glucose metabolism have not been fully elucidated, most recent evidence from animal models suggests that improvement in insulin sensitivity may underlie its hypoglycemic effects [13]. It is not known whether ginseng can reverse insulin resistance induced by PIs.

Herbals treatments, like ginseng, are perceived to be safe by patients, but pharmacologic interactions with concomitant conventional drugs are a major concern. Ginseng was recently found to reduce the anticoagulant effect of warfarin [14]. However, its potential interaction with antiretrovirals has not been investigated. Other herbs, such as St. Johns' wort [15] and garlic [16] dramatically reduce the concentrations of PIs in healthy volunteers, in the case of St. John's wort, through the induction of their common metabolic pathway, cytochrome P450 3A4 (CYP450 3A4). This could lead to a decrease in effectiveness and potentially result in treatment failure.

The goals of this study were to evaluate potential PK interactions between IDV and AG, and assess whether AG improves IDV-induced insulin resistance.

## Methods

### Study Participants

Healthy volunteers, male and female, 18–64 years of age, were recruited through newspaper advertisement, flyers and word of mouth. Exclusion criteria included a history of nephrolithiasis, use of any prescription medications, over the counter medications, or dietary supplement within 14 days of enrollment, body mass index (BMI, weight (kg)/height (m^2^)) ≥ 30 kg/m^2^, or blood donation within 30 days of study enrollment. Prior to enrollment, subjects were required to have a negative HIV-1 antibody test, normal values for serum creatinine, aspartate aminotransferase (AST), alanine aminotransferase (ALT), and hemoglobin, and a normal physical examination. The study was approved by the Institutional Review Board at Johns Hopkins University School of Medicine and all participants signed informed consent.

### Study Design

After initial screening, participants were admitted to the Johns Hopkins inpatient General Clinical Research Center (GCRC) on three separate occasions over a 21 day period for PK and metabolic studies. During the first inpatient visit, baseline insulin sensitivity was assessed. Subjects were then discharged with instructions to take IDV 800 mg by mouth (Crixivan, Merck and Company, Rahway, New Jersey, USA) every 8 hours (0600, 1400, 2200) on an empty stomach beginning three days before the subsequent inpatient visit. During the second inpatient visit, insulin sensitivity was reassessed and an 8-hour PK IDV sampling was obtained. Subjects were then instructed to take IDV, as taken previously, and to co-administer encapsulated AG ground root, 1 gram by mouth every 8 hours. Outpatient safety visits with laboratory assessments occurred 7 days before and 7 days after the final inpatient visit.

Dried whole-root AG was obtained from a single batch through the Wisconsin Ginseng Board (Wausau, Wisconsin). The identity of the dried whole root of AG was verified by the Wisconsin ginseng Board prior to encapsulation. Following verification the dried whole-root AG was ground and encapsulated in 500 mg capsules by the American Pharmaceutical Nutraceutical Laboratories (Wausau, Wisconsin) and dispensed by the Johns Hopkins Hospital Investigational Drug service. AG was administered under Investigational New Drug Application # 69,866 granted by the Food and Drug Administration. The dose of AG was selected based on previous studies investigating the effect of AG on glucose tolerance in humans[12,17]. The dose of IDV was selected according to its package insert. Following 14 days of IDV and AG, subjects returned to the inpatient GCRC for reassessment of insulin sensitivity and IDV PK.

### Ginseng Analyses

The concentrations of six common ginsenosides (Rg1, Re, Rb1, Rc, Rb2, and Rd) were measured, following procedures described by Chuang [18], in the Analytical Laboratory of the Johns Hopkins University Division of Clinical Pharmacology. Briefly, five different capsules were randomly selected from five separate bottles. The content of each capsule was extracted with 3 mL of ethanol:water (50:50, vol:vol), sonicated, and centrifuged. The pellet was extracted two more times and the resulting liquid was filtered. Samples and ginsenoside standards (Sigma-Aldrich, St. Louis, Missouri) were analyzed by High Performance Liquid Chromatography (HPLC) using Beckman Ultrasphere 5 μm, 250 × 4.6 mm column. The amount of each ginsenoside assayed in the capsules was as follows (expressed as percent per gram of ginseng): Rg1 0.11, Re 1.06, Rb1 0.91, Rc 0.08, Rb2 0.19, Rd 0.08, Total ginsenosides 2.43. The intra- and interassay coefficients of variation (CV) were < 10.5% and < 8.5%, respectively with > 85% accuracy.

### Bioassay for Hypoglycemic Activity

The hypoglycemic activity of the batch of AG used in the study was assessed in diabetic mice prior to initiation of the clinical studies, as previously described [13]. Briefly, male *ob/ob *mice (n = 6) received AG extract dissolved in distilled water by daily intraperitoneal injection at a dose of 250 mg/kg. Fasting serum glucose was measured from blood obtained from tail samples at baseline, day 5 and day 12. The hypoglycemic response in AG-treated mice was compared to that observed in *ob/ob *mice who received vehicle following the same protocol. Animals were fed rodent chow and housed in environmentally stable conditions in metabolic cages. Food consumption and weight were monitored daily. All animal experiments were carried out at the Tang Center for Herbal Medicine Research, University of Chicago in the laboratory of Chun-Su Yuan, MD, PhD.

### PK Sampling and Analysis

During the second and third inpatient GCRC visits, nine blood samples were collected immediately before administration of the morning dose of IDV and at 0.5, 1,2,3,4,5,6, and 8 hours post-dose after an overnight fast. The previous observed dose was given at 2200. PK data were analyzed using non-compartmental methods using WinNonlin^® ^(Pharsight, Cary, NC). IDV PK parameter estimates included area under the plasma-concentration-time curve from time 0 to 8 hours after the dose, (AUC_0–8 hrs_), maximum plasma concentration (C_max_), minimum plasma concentration (C_min_), and time to maximum plasma concentration (T_max_).

### Metabolic Assessments

Body composition, including fat-free mass (FFM), was assessed at baseline using dual x-ray absorptiometry (Hologic-QDR 4500W, Hologic Co, Bedford, MA, intra-subject CV < 2%). BMI was assessed during each inpatient admission and was calculated as the weight in kilograms/height in meters squared.

Peripheral sensitivity to glucose was assessed by the hyperinsulinemic euglycemic clamp technique [19]. Subjects were admitted to the GCRC in the afternoon prior to the clamp procedure and maintained on a eucaloric diet (50% carbohydrate, 30% fat, and 20% protein). After an overnight fast, an intravenous catheter was placed in the antecubital vein for infusion of glucose and insulin. A second catheter for blood sampling was inserted in a dorsal vein of the hand in a retrograde fashion. The hand was placed into a 50°C warming box to "arterialize" the samples [20]. After baseline blood samples were obtained, a primed, continuous infusion of regular insulin (40 mU/m^2^/min) was started, followed by a constant infusion of 20% dextrose at a variable rate, based on 5-minute measurements of blood glucose, in order to maintain blood glucose concentrations at each subject's baseline level ± 5%. Infusion continued for a total of 180 minutes.

Glucose utilization (M) was calculated as the average glucose infusion rate (milligrams of glucose/kilograms of FFM/minute) during the last 60 minutes of the infusion (i.e. steady state portion). FFM was derived from the baseline evaluation by dual energy X-ray absorptiometry (DXA).

During the final 60 minutes of the insulin clamp procedure, samples were obtained at 10 minutes intervals to determine insulin concentrations. M divided by the average insulin concentration in the final 60 minutes (I), which represents the amount of glucose metabolized per unit of insulin, was used as the primary measure of insulin sensitivity. During the second and third inpatient visits, the morning dose of IDV (and AG for the third inpatient visit) was given immediately prior to the beginning of the insulin clamp procedure. Fasting serum glucose and homeostasis model assessment of insulin resistance (HOMA-IR), a widely used marker of insulin sensitivity [21], were assessed prior to the insulin clamp procedure at each of the inpatient study visits.

The serum glucose concentration during the final 60 minutes of the clamp was 89.8 ± 1.0 mg/dL which was 97.8 ± 0.3% of the targeted glucose concentration with a coefficient of variation of 4.2 ± 0.3% (n = 32 clamp studies). The average insulin concentration during the final 60 minutes of the infusion was 69.2 ± 1.7 μU/mL.

### Measurements

Serum glucose was measured using the glucose oxidase method (Beckman Instruments, Fullerton, CA). Serum insulin concentrations were determined by enzyme immunoassay, using a TOSOH 1800 (Tosoh Corporation, Tokyo, Japan). Intra-assay and inter-assay CVs range from 1.4–2.3% and 5–6%, respectively. IDV concentrations were measured by HPLC-mass spectrometry. The intra- and inter-assay CVs were < 9% and < 8%, respectively with > 94% accuracy.

### Statistical Calculations

Paired comparisons between outcome measures obtained during the first and second inpatients visits (i.e. Baseline vs. IDV Condition) and during the second and third inpatient visits (IDV vs IDV + AG) were made using a paired t-test. Univariate relationships between continuous variables were assessed with linear regression. All data are presented as mean ± standard error of the mean (SEM). Differences were regarded as statistically significant if p < 0.05. All data analysis was performed using Stata (Version 8.1, Stata Corporation, College Station, TX). Pharmacokinetic parameter estimates were summarized using geometric means with 95% confidence intervals; values obtained with and without AG were compared using a geometric mean ratio and 90% confidence interval.

## Results

### Preclinical Studies

**Figure 1:**
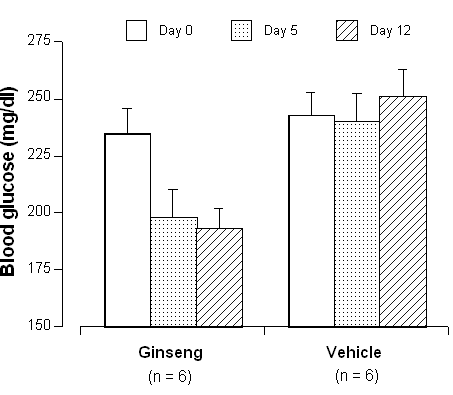
Fasting Glucose Concentrations in Leptin-Deficient (*ob/ob*) Mice Treated with AG-Extract (250 mg/kg) and Vehicle Over 12 Days (n = 6 in both groups).

In *ob/ob *mice receiving AG extract, fasting glucose concentrations decreased (Baseline vs Day 12; 235 ± 11 mg/dL vs 193 ± 9 mg/dL, between group t-test, p = 0.003), but remained constant in the vehicle-treated group (243 ± 10 mg/dL vs 251 ± 12 mg/dL) (Figure 1). Weight did not change over the study interval in the AG-treated mice (data not shown).

### Clinical Studies

Fourteen healthy volunteers were evaluated (Table 1). All participants were male, 12 were African-American, and two were white. Age ranged from 26–53 years with a mean of 42.9 ± 1.9. The average BMI was 26.1 ± 0.8 kg/m^2 ^(range 21.9–29.9).

**Table 1:** Demographic Characteristics of Study Participants

**Subject #**	**Race**	**Age (years)**	**BMI (kg/m^2^)**	**Fat (%)**
1	AA	41	29.9	29.5
2	AA	43	22.4	12.2
3	AA	41	22.3	12.6
4	AA	53	26.6	23.0
5	W	26	29.0	25.2
6	AA	49	29.9	32.1
7	AA	43	24.7	22.3
8	AA	44	21.9	21.1
9	AA	36	29.1	29.9
10	AA	37	24.0	24.7
11	AA	52	23.4	15.8
12	AA	42	29.3	19.7
13	AA	46	26.7	26.7
14	W	49	26.5	26.1

AA – African-American; W-White

#### PK Analysis

Thirteen participants were included in the analysis of the comparison of IDV PK with and without AG. Subject 9 was noted to develop transaminitis after the second inpatient study visit and did not complete the remainder of the study. Table 2 shows the average IDV PK parameters after 3 days of IDV following the morning dose, and after co-administration of 14 days of IDV and AG. All PK parameters were similar in the two periods.

**Table 2:** PK parameter estimates of IDV before and after co-administration with AG (1 gram every 8 hours), n = 13

	**IDV alone (Day 7) ** **GM (95%CI)**	**IDV + AG (Day 22) ** **GM (95%CI)**	**Day 7/Day 22 ** **Geometric Mean Ratio ** **(90% CI)**
**C_max _(ng/mL)**	5623 (4027–7853)	5633 (4908–6464)	1.00 (0.77–1.29)
**C_min _(ng/mL)**	61.97 (42.72–89.88)	62.78 (39.72–99.24)	1.00 (0.77–1.29)
**t_max _(hours)**	0.98 (0.7189–1.3403)	0.74 (0.5527–0.9870)	0.75 (0.53–1.06)
**AUC_0–8 _(ng·h/dL)**	12171 (8628–17168)	10700 (8515–13649)	0.89 (0.72–1.08)

#### Metabolic Studies

##### Insulin Sensitivity: Effect of IDV

Eleven subjects were included in the analysis to assess the effect of IDV on insulin sensitivity. The calculated M and M/I values for subject 4–14 is presented in Table 3. The data from subjects 1–3 were excluded since the protocol was changed after these subjects completed their participation in protocol. The protocol was changed to better match peak IDV concentrations with the steady state portion of hyperinsulinemia during the insulin clamp. Specifically, for subjects 4 – 14, the morning dose of IDV was administered immediately prior to the beginning of the insulin clamp procedure, rather than two hours before the start of the insulin clamp as originally planned and executed for subjects 1 – 3.

**Table 3:** Glucose Utilization (M) and Insulin  Sensitivity (M/I) Measured by the Hyperinsulinemic Euglycemic Clamp in  Healthy Volunteers at Baseline, After 3 Days of IDV, and After  Co-Administration of IDV and AG (1 gram every 8 hours) for 14 Days

**Subject**	**M ** (mg/kg FFM/min)			**M/I ** (mg/kg FFM/min per μU/ml of insulin)		
	**Baseline**	**IDV Alone**	**IDV + AG**	**Baseline**	**IDV Alone**	**IDV + AG**

**4**	8.25	5.69	5.79	0.121	0.072	0.072
**5**	8.46	8.20	10.35	0.135	0.138	0.193
**6**	4.58	3.93	4.01	0.055	0.049	0.059
**7**	11.52	7.14	7.82	0.173	0.141	0.134
**8**	8.88	9.02	7.29	0.132	0.155	0.118
**9**	4.10	2.74	-	0.062	0.037	-
**10**	10.14	9.54	9.94	0.119	0.126	0.123
**11**	9.60	8.62	5.93	0.157	0.115	0.107
**12**	6.19	4.24	3.51	0.078	0.062	0.042
**13**	8.62	7.82	7.66	0.137	0.123	0.113
**14**	4.60	2.62	2.37	0.068	0.038	0.030

**Figure 2:**
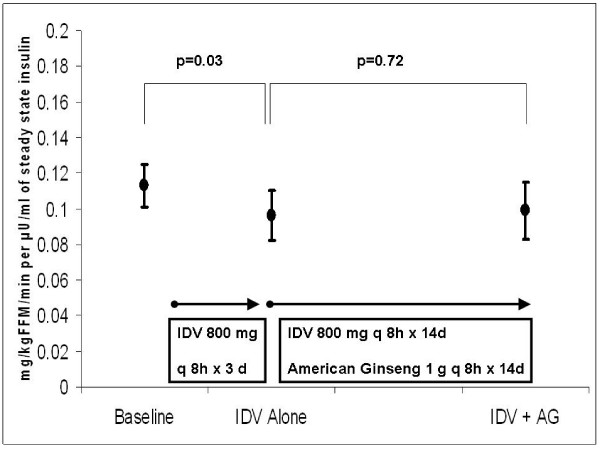
Insulin Sensitivity (Glucose Infusion Rate Per Kilogram of Free-Fat Mass Per Unit of Insulin) at Baseline, After 3 Days of Indinavir, and After 14 Days of Co-Administration of AG in Healthy Volunteers (p-values represent the average mean difference by paired t-test).

Compared to baseline measurements, IDV was associated with an average decrease in insulin sensitivity (M/I) of 14.8 ± 5.9%, from 0.113 ± 0.012 to 0.096 ± 0.014 mg/kg FFM/min per μU/ml of insulin, p = 0.03) (Figure 2). We also detected a decrease in average glucose utilization (M) with IDV administration when compared to baseline (from 7.73 ± 0.75 to 6.32 ± 0.78 mg/kg FFM/min, p = 0.005). Fasting glucose and HOMA-IR did not change when comparing baseline to the second admission (92.4 ± 1.8 mg/dL vs 95.0 ± 2.4 mg/dL, p = 0.27; 1.34 ± 0.32 vs 1.34 ± 0.23, p = 0.97, respectively).

##### Insulin Sensitivity: Effect of AG

Ten subjects were included to assess the effect of AG on IDV-induced insulin resistance (Table 3). One subject (subject 9) did not have complete data for this analysis because he was discontinued from the study after the second insulin clamp procedure, as previously noted. There was no difference in insulin sensitivity between the second (IDV alone) and third (IDV + AG) insulin clamp procedures, 0.102 ± 0.013 vs 0.099 ± 0.016 mg/kg FFM/min per μU/ml of insulin, respectively (p = 0.72) (Figure 2). Glucose utilization (M) with IDV administration also did not change when AG was co-administered, 6.68 ± 0.76 vs 6.47 ± 0.84 mg/kg FFM/min, respectively (p = 0.62).

Fasting glucose and HOMA-IR did not change after 2 weeks of AG (93.0 ± 1.4 mg/dL vs 92.0 ± 2.6 mg/dL, p = 0.65; 1.4± 0.25 vs 1.4 ± 0.28, p = 0.95). Body weight did not change during the trial (baseline vs third inpatient study visit, 83.1 ± 3.1 kg vs 83.2 ± 3.1 kg, p = 0.88).

##### Insulin Sensitivity: Effect of AG after Normalization for IDV Concentrations

Because of the variability of IDV concentrations with and without co-administration of AG, we evaluated the potential effect of AG on insulin sensitivity after adjustment for IDV concentrations. In a *post hoc *analysis, we standardized the measurement of insulin sensitivity for IDV concentrations by dividing M/I for the second and third insulin clamp procedures by the corresponding IDV AUC between 2–3 hours (AUC_2–3_). We then multiplied this value by 10^6 ^for ease of interpretation.

We chose the 2–3 hour IDV AUC since it is the same time period during the clamp procedure in which insulin sensitivity is assessed and there was a trend toward correlation between insulin sensitivity (M/I) and IDV AUC_2–3 _during the third insulin clamp procedure (r = 0.58, p = 0.078). The IDV AUC_2–3 _was not correlated with M/I (r = 0.04, p = 0.90) during the second clamp procedure (IDV alone). No other IDV PK parameters or other fractional AUCs were correlated with M/I for either the second or third clamp procedure (data not shown).

For the 10 subjects with measurable M/I estimates normalized for IDV AUC_2–3_, the average difference in IDV AUC_2–3 _was similar when comparing the period of IDV alone to the period with concomitant IDV and AG (2353 ± 389 vs 1923 ± 483 ng·hr/mL; difference: 430 ± 330 ng·hr/mL, p = 0.22). When each subject's M/I was divided by the IDV AUC_2–3_, which can be interpreted as insulin sensitivity per unit of plasma IDV, a significant increase was seen after AG co-administration (60.9 ± 15.4 vs 92.2 ± 20.9 mg Glucose·10^6^/kg FFM/min/μU/ml of insulin/ng·hr/mL IDV, p = 0.03). A similar increase was seen when comparing M (multiplied times 10^6^) divided by the IDV AUC_2–3 _between the IDV alone period and the concomitant IDV plus AG period (4240.6 ± 1180.0 vs 6100.5 ± 1526.8 mg Glucose·10^6^/kg FFM/min/ng·hr/mL IDV, p = 0.05).

##### Safety Assessment

Both IDV and AG were well tolerated. There were no serious adverse events. Three subjects developed transaminitis. In one subject (as previously mentioned), an AST elevation 3 times the upper limit of normal (Grade 2) after 3 days of IDV required study discontinuation. Three subjects were noted to have mild elevations in bilirubin. One subject had a mild increase in serum creatinine. One patient reported an episode of vomiting and one subject reported dyspepsia after dose administration. All laboratory abnormalities and symptoms normalized after discontinuation of the study drugs. With the exception of the Grade 2 transaminites, all adverse events occurred during the co-administration of IDV and AG.

##### Adherence Assessment

By pill count assessed on three occasions during the study (i.e., the second and third inpatient visit and the intervening outpatient safety visit), IDV overall mean adherence was 97.7 ± 1.5%. AG adherence, as assessed on two occasions (interim outpatient visit and visit three), was 96.6 ± 2.5%.

## Discussion

In this prospective study of healthy volunteers, we found that 14 days of co-administration of AG did not significantly impact IDV PK. We also found that, although IDV acutely reduced insulin sensitivity by an average of 15%, AG did not change insulin sensitivity in IDV-treated healthy volunteers, providing evidence against its use in the treatment of PI-induced disorders of glucose metabolism.

Potential drug-herb interactions are an important safety concern for many clinicians and HIV-infected patients. Although herbal remedies are perceived as safe by many patients, some can inhibit and/or induce the CYP3A4 enzyme, the main metabolic pathway for most PIs and non-nucleoside reverse transcriptase inhibitors (NNRTIs), potentially leading to increased toxicity or therapeutic failure [15,16]. The metabolic pathways of ginseng components are not well known. Ginsenosides, which are steroid-like molecules of the saponin class, are thought to be the active compounds of all ginseng species [22]. There are more than 20 different types of ginsenosides in ginseng root and their relative concentrations varies depending on the species, the batch, and the part of the plant assayed (eg. root vs berry)[23,24]. The ginsenosides also differ in their effects on metabolism. In *in vitro *models investigating the catalytic activity of c-DNA expressed CYP450 isoforms, the ginsenoside, Rd, has been shown to be a weak inhibitor of CYP3A4, while Rf increased CYP3A4 activity by 54% [25]. In addition, metabolites of ginsenosides as well as non-ginsenoside components of ginseng have also been shown to inhibit CYP3A4 in experimental models [26,27]. The clinical significance of these *in vitro *observations is not clear.

In previous human studies, Siberian ginseng (*Eleutherococcus senticosus*) has been shown to increase concentrations of nifedipine [28], a CYP3A4 substrate, but does not affect concentrations of midazolam [29], another CYP3A4 probe drug. It should be noted that Siberian ginseng is not a species of the genus *Panax*, contains no ginsenosides, and therefore the effects of Siberian ginseng are not generalizable to members of the *Panax *genus, such as AG. However, human studies in healthy volunteers using *Panax ginseng *(C.A. Meyer) have shown no interaction with midazolam [30] and no change in urinary 6-beta-OH-cortisol/cortisol ratio [31], both measures of CYP3A4 activity.

We also found that 3 days of IDV administration decreased insulin sensitivity by 15% in healthy volunteers. Our findings are similar to another healthy volunteer study, showing a 17% decrease in insulin sensitivity with IDV administration (800 mg q 8 hours)[32], but smaller in magnitude when compared to another study using a higher dose (34% decrease with a single dose of 1200 mg) [33]. In *in vitro *studies and animal models, PIs, such as IDV, impede glucose movement through the major glucose transporter in skeletal muscle, GLUT4, thereby inducing insulin resistance[34]. The observations in humans, including ours, are consistent with this mechanism, although the extent to which it is clinically relevant in the pathogenesis of hyperglycemia among HIV-infected, HAART-treated patients remains unclear.

Although we observed that insulin sensitivity decreased with IDV administration, we did not find any change in insulin sensitivity with co-administration of AG. In previous studies, Vuksan, *et al*. showed that AG administration immediately prior to an oral glucose tolerance test was associated with 20% decrease in glucose AUC in both patients with type 2 diabetes mellitus and healthy volunteers [35], but this effect appears to be dependent on the batch of ginseng used [23]. To rule out an inert batch of AG, we conducted a bioassay of our batch using an *ob/ob *mouse model and a hypoglycemic effect was observed, suggesting that our batch possessed some active components.

The mechanism underlying the previously observed effects of AG on glucose metabolism remains unknown and may be multifactorial. The modulation of digestion and enhancement of insulin secretion have been proposed based on findings in some animals models [36,37], but ginseng administration has also been shown to improve insulin sensitivity in *ob/ob *mice by more than two-fold [13]. It has been postulated that ginsenosides may intercalate into the cellular plasma membrane, thus modulating the cell signaling, electrolyte transport, and receptor binding [22]. It is not known whether this effect could also modulate the effect of IDV on the glucose flux through the GLUT4 transporter.

Another factor that may have contributed to the lack of effect is the variability of IDV plasma concentrations between the two clamps, which has been previously observed [38]. The effect of AG on IDV-induced insulin resistance would be best determined if IDV plasma concentrations were the same when it was given alone and when it was co-administered with AG. For this reason, we normalized the measure of insulin sensitivity for drug concentration in a *post hoc *analysis, by dividing M/I by the IDV concentration during the steady state portion of the clamp and found that this ratio was significantly higher in the IDV + AG condition.

Although the interpretation of ratios can be challenging [39] and should be done with caution, one explanation of this finding is that insulin sensitivity per unit of IDV concentration modestly improved with the administration of AG. Given the known effect of IDV on GLUT4 blockade, AG components may work directly at the plasma membrane to allow glucose to enter cells, either by improving glucose movement through GLUT4, increasing the concentration of GLUT4 in the plasma membrane, or facilitating glucose entry through alternate pathways. Another possible mechanism of AGs effect may be through the enhancement of local blood flow. Increased capillary recruitment mediated though nitric oxide is an important mechanism of increasing glucose and insulin delivery to skeletal muscle [40,41]. IDV has been shown to cause endothelial dysfunction in healthy volunteers, likely by reducing nitric oxide production [42]. Ginseng species have been shown in experimental models to increase nitric oxide production [43,44] and therefore, may effect insulin sensitivity by this mechanism. This hypothesis requires further investigation.

Our study had additional limitations which may have implications for the generalizability of its findings. Although both men and women were eligible for participation, only men enrolled. In previous studies, inducibility of CYP3A4 by herbal compounds has shown an interaction by sex, whereby women showed a 74% increased effect of St. John's wort on CYP3A4 activity using a midazolam probe compared to men [45]. In the same study, however, *Panax ginseng *showed no effect on the midazolam metabolism in either men or women. Further PK interaction studies in women may be necessary. In addition, our population was mostly African-American. Although there is evidence for genotypic differences in CYP3A4 between Caucasians and African-Americans, phenotypic differences in CYP3A4 activity have not been found [46]. Finally, although we quantified the amount of common ginsenosides, there is substantial variability of composition even within species. As a result, as is the case in all botanical research using non-standardized complex compounds whose active component is unknown, the extent to which our findings are generalizable to other AG products is not clear.

In conclusion, this study in healthy volunteers found no evidence of a significant PK interaction between AG and IDV. Because CYP3A4 is the principal metabolic pathway of most HIV PIs and NNRTIs, significant drug-herb interactions with these antiretrovirals are unlikely. The metabolic effects of AG are more difficult to interpret. Although IDV significantly reduced insulin sensitivity, there was no change in insulin sensitivity with AG administration. However, after normalization for IDV concentrations, insulin sensitivity improved in the AG condition, leaving open the possibility of a modest effect on glucose metabolism. Further studies to clarify this question should attempt to reduce variability in IDV plasma concentrations, either by giving multiple doses of IDV at shorter intervals to maintain plasma IDV concentrations constant while insulin sensitivity is assessed, or using an IDV regimen boosted with ritonavir. Until these issues are resolved, there is no clear scientific basis to recommend AG for the treatment of glucose abnormalities in HIV-infected patients.

## Competing interests

Drs. Dobs, Parsons, Caballero, and Yuan declared that they have no competing interests. Dr. Hendrix received research support from Merck and Company. Dr. Andrade served as a consultant for Abbott Laboratories. Dr. Brown served as a consultant or has received research support from Merck and Company, Abbott Laboratories, Reliant Pharmaceuticals, Glaxo Smith Kline, EMD-Serono, and Theratechnologies. Dr. Flexner served on a Scientific Advisory Board for Merck and Company.

## Authors' contributions

TTB performed the insulin clamps. C–SY carried out the animal studies. TLP carried out the measurements of ginsenoside and IDV plasma concentrations. TTB and ASAA drafted the manuscript. ASAA, TTB, CWF, CH C–SY, ASD and BC participated in the design of the study. TTB, ASAA, and CH performed the statistical analysis. ASAA, TTB, ASD, CH and BC participated in the data interpretation. TTB and ASAA conceived of the study and participated in its coordination. All authors read and approved the final manuscript.

## Pre-publication history

The pre-publication history for this paper can be accessed here:


